# Shift work as an oxidative stressor

**DOI:** 10.1186/1740-3391-3-15

**Published:** 2005-12-28

**Authors:** Akbar Sharifian, Saeed Farahani, Parvin Pasalar, Marjan Gharavi, Omid Aminian

**Affiliations:** 1 Department of Occupational Medicine, Tehran University of Medical Sciences, Tehran, Iran; 2 Department of Medical Biochemistry, Tehran University of Medical Sciences, Tehran, Iran; 3 Private practice, Tehran, Iran

## Abstract

**Background:**

Some medical disorders have higher prevalence in shift workers than others. This study was designed to evaluate the effect of night-shift-working on total plasma antioxidant capacity, with respect to the causative role of oxidative stress in induction of some of these disorders.

**Methods:**

Two blood samples were taken from 44 workers with a rotational shift schedule, one after their day shift and one after their night shift. The total plasma antioxidant capacity of each worker was measured through the FRAP method. The impacts of age and weight were also assessed.

**Results:**

The total plasma antioxidant capacity was measured in 44 shift-workers with a mean age of 36.57 years (SD: 10.18) and mean BMI of 26.06 (SD: 4.37) after their day and night shifts. The mean reduction of total plasma antioxidant capacity after the night shift was 105.8 μmol/L (SD: 146.39). Also, a significant correlation was shown between age and weight and total plasma antioxidant capacity. Age and weight were found to be inversely related to total plasma antioxidant capacity; as age and weight increased, the total plasma antioxidant capacity decreased.

**Conclusion:**

Shift work can act as an oxidative stressor and may induce many medical disorders. Aging and obesity in shift workers makes them more sensitive to this hazardous effect.

## Introduction

Shift work is defined as work primarily outside of normal daytime working hours [1,2]. Nowadays the number of shift workers has increased due to technological development. Shift work is accompanied by a greater incidence of many medical disorders, such as cardiovascular, gastro-intestinal, and neurological disorders [3,4].

The biological mechanisms that show how shift work acts to induce such disorders in workers are relatively unknown. The known mechanism that induces and promotes cellular damage and results in such disorders is known as oxidative stress. The antioxidant system is the defense system that neutralizes the free radicals produced in the hazardous oxidative pathway. When the production of free radicals exceeds body antioxidant capacity, oxidative stress occurs [5-8].

This study was designed to investigate whether shift work acts as an oxidative stressor. Total plasma antioxidant capacity was measured as an indicator of oxidative stress in shift workers. Factors that may influence antioxidant capacity, such age and weight, were also measured.

## Methods

This study was performed in an industrial catering with 220 male personnel with a rotational (day-night-off-off) working schedule. Only non-smokers without known chronic diseases (HTN, DM, CVD, IHD, CRF, hepatitis) were selected for study. Workers with acute diseases during the study were excluded from data analysis.

Two blood samples were taken from each participant, one after their day shift and one after their night shift. The blood samples were mixed with EDTA and frozen at -20°C. The total serum antioxidant capacity was measured by the FRAP method. All participants were fast in the last 5 hours of their shift. The mean of total plasma antioxidant capacity at the end of day work was compared with the mean of total plasma antioxidant capacity at the end of night work by means of a paired t test.

Age, weight, and height of participants were determined in order to evaluate the influence of these parameters on antioxidant capacity. Impacts of age and BMI on total plasma antioxidant capacity were calculated by means of a Pearson correlation.

Statistical analysis was conducted by means of SPSS statistical soft ware (version 11), a paired t-test, and Pearson correlation. A p value of less than 0.05 was considered to be significant.

## Results

**Figure 1:**
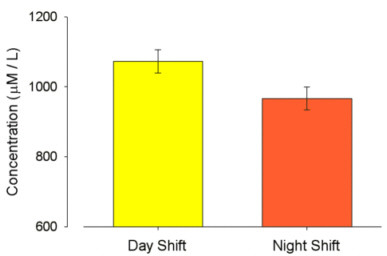
Mean total plasma antioxidant capacity during day shift and night shift.

Forty-four shift workers with a mean age of 36.75 yrs (SD: 10.18) completed the study. The mean total plasma antioxidant capacities after day shift and night shift are shown in Figure 1. There was a mean reduction of total plasma antioxidant capacity from the day to the night shift of 105.8 μmol/L (SD: 146.39), p < 0.001.

**Figure 2:**
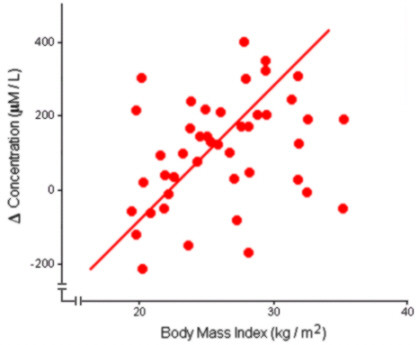
**Total plasma antioxidant capacity as a function of body mass index**. Linear regression is indicated by the straight line (r = 0.314, p = 0.019).

The correlation between age and total plasma antioxidant capacity was found through a Pearson correlation: r = 0.253, p = 0.049. The correlation between BMI and total plasma antioxidant capacity was also determined by means of a Pearson correlation: r = 0.314, p = 0.019 (Figure 2).

## Discussion

Oxidative stress is known to result in several acute and chronic disorders [5-8], but the factors that induce and promote this condition are variable. Oxidative stress occurs when the production of free radicals exceeds the defensive response of the antioxidant system. Oxidative stress has a major role in the causality of some disorders that have higher prevalence in shift workers, such as cardiovascular disorders [4].

The hypothesis was that shift work would act as an oxidative stress. This study was designed to test this hypothesis. The total plasma antioxidant capacity was measured as an indicator of oxidative stress occurrence in shift workers. The effect of age and weight on total plasma antioxidant capacity was also assessed. The results of this study show that shift work can act as an oxidative stressor and, as age and BMI rise, the antioxidant system becomes more disabled against oxidative stress. A special dietary regimen including antioxidant agents, such as vitamins, may be beneficial to shift workers.

## Conclusion

Shift work can act as an oxidative stressor. A special dietary regimen including antioxidant agents, such as vitamins, may be beneficial to shift workers.

## Competing interests

The author(s) declare that they have no competing interests.

## Authors' contributions

**SA **participated in the design of the study and performed and coordinated it.

**FS **participated in its design and the data collection.

**PP **conceived of the study and helped with its coordination.

**GM **performed the statistical analysis.

**AO **drafted the manuscript.

All authors read and approved the final manuscript.

## References

[B1] Pati AK, Chandrawshi A, Reinberg A (2002). Shift work: consequence and management. Curr Sci.

[B2] Husbands R, Schneider G, Li S (1995). Working time around the world (Condition of Work Digest Volume 14).

[B3] Williams C (1998). Social factors, work, stress and cardiovascular disease prevention in the European Union.

[B4] Sjoblom TS, Kalimo R (1997). Shift work, occupation and coronary disease – over 6 years of fallow up in the Helsinki heart study. Scand J Work Environ Health.

[B5] Gutteridge JMC (1995). Lipid proxidation and antioxidants as biomarkers of tissue damage. Clinchem.

[B6] Punchard NA, Kelly FJ (1996). Free Radicals.

[B7] Jial I, Fuller CJ (1993). Oxidized LDL and antioxidant. Clin Cardiol.

[B8] Markesbery WR (1999). Oxidative alterations in Alzheimer's disease. Brain Pathol.

